# Personality Dimensions of Subjects With Aviophobia: A Case-Control Comparison With Frequent Fliers

**DOI:** 10.7759/cureus.78411

**Published:** 2025-02-03

**Authors:** Piercarlo Minoretti, Giovanni Fortuna, Davide D’Acquino, Konstantinos Lavdas

**Affiliations:** 1 Occupational Health, Studio Minoretti, Oggiono, ITA

**Keywords:** agreeableness, air travel, aviophobia, conscientiousness, five-factor model, neuroticism, personality dimensions

## Abstract

Background: Aviophobia (AP), or fear of flying, is a common situational phobia that can profoundly impact an individual’s personal, social, and professional life. Investigating personality traits through the framework of the five-factor model (FFM) and their relationship to AP may offer valuable insights with potential applications in preventive and therapeutic interventions. Here, we conducted a case-control analysis comparing the “big five” personality dimensions between individuals with AP and frequent fliers (FFs). We specifically selected FFs as controls due to their demonstrated ability to adapt to aviation-related scenarios and regular exposure to flight environments.

Methods: We included 60 subjects who met the DSM-5 criteria for specific phobia (situational type: flying), matched in a 1:1 ratio, with 60 FFs serving as controls. Personality traits were assessed using the NEO Five-Factor Inventory (NEO-FFI), a validated 60-item instrument that evaluates five core personality dimensions: neuroticism, extraversion, conscientiousness, agreeableness, and openness. Multivariable logistic regression analysis was performed to explore independent associations between the “big five” personality dimensions and the presence of AP.

Results: Subjects with AP exhibited significantly higher neuroticism scores (36.2 versus* *30.8, p < 0.001) and lower scores for conscientiousness (40.7 versus* *45.4, p < 0.001) and agreeableness (37.4 versus* *42.2, p < 0.001) compared to FFs. No significant differences were observed with respect to extraversion or openness. In a fully adjusted multivariable model, neuroticism (OR = 1.14, 95% CI = 1.05-1.23, p < 0.001), conscientiousness (OR = 0.85, 95% CI = 0.77-0.93, p < 0.001), and agreeableness (OR = 0.87, 95% CI = 0.79-0.94, P < 0.01) remained independently associated with AP.

Conclusions: We identified distinct personality profiles among individuals with AP compared to FFs, characterized by elevated neuroticism and diminished conscientiousness and agreeableness. The current findings may enhance our understanding of the psychological mechanisms underlying flight-related anxiety and suggest the value of personality-based approaches in developing targeted interventions for AP.

## Introduction

Aviophobia (AP), a specific form of situational phobia also known as fear of flying, represents a clinically significant psychological condition characterized by an excessive and persistent fear or anxiety specifically associated with air travel, persisting for a minimum of six months and distinct from other mental health conditions [1-3]. This complex psychological phenomenon manifests primarily through pronounced avoidance behaviors, wherein affected individuals either completely abstain from air travel or experience severe psychological distress during flights [4,5]. The epidemiological landscape of AP exhibits considerable variability, with prevalence rates ranging from 2.5% to 40%, contingent upon diagnostic methodologies and study populations [6,7]. The ramifications of AP extend beyond individual psychological well-being, encompassing both personal and socioeconomic dimensions [7]. At the individual level, AP can precipitate profound emotional distress, heightened anxiety states, and maladaptive avoidance patterns, substantially compromising quality of life and interpersonal relationships [8]. Furthermore, this condition can significantly constrain professional advancement opportunities, educational pursuits, and social engagement by limiting geographical mobility [9]. Importantly, fear of flying has documented impacts on airline revenues and passenger behavior, with historical studies indicating meaningful financial implications for carriers [10]. Consequently, advancing our understanding of AP can help inform both therapeutic approaches for individuals and airline service strategies.

The exploration of personality traits through the lens of the five-factor model (FFM) [11] and their association with AP development represents an intriguing area of research with significant implications for preventive interventions and therapeutic strategies. The FFM conceptualizes personality along five fundamental dimensions, collectively known as the “big five” [11,12]: neuroticism (characterized by emotional lability and anxiety predisposition), extraversion (manifested through sociability and outward-directed behavior), conscientiousness (reflected in organizational aptitude and efficiency), agreeableness (expressed through interpersonal warmth and empathic tendencies), and openness (demonstrated by intellectual curiosity and creative disposition). A growing body of research has demonstrated strong associations between hierarchically structured personality dimensions and a range of psychopathological manifestations. For instance, high neuroticism is closely linked to mood and anxiety disorders, low extraversion is associated with social phobia and dysthymia, low conscientiousness correlates with substance use disorders, and low agreeableness is tied to antisocial and narcissistic personality disorders [13,14]. These findings underscore the utility of the FFM as a valuable theoretical framework for exploring the underlying mechanisms of specific phobias [15-17].

Our study, based on the FFM, investigated whether individuals with AP exhibit characteristic personality patterns. Specifically, we hypothesized that, compared to frequent flyers (FFs), aviophobic individuals would show higher neuroticism scores while scoring lower on extraversion, openness to experience, conscientiousness, and agreeableness. We selected FFs as our control group due to their proven ability to cope with air travel, enabling a meaningful comparison of the “big five” personality dimensions.

## Materials and methods

Study participants

This case-control study was conducted between June 2022 and June 2024 at Studio Minoretti srl (Oggiono, Italy). We employed convenience sampling through targeted advertisements to recruit adults (≥18 years) who utilized air transportation. AP was defined according to the DSM-5 criteria for specific phobia (situational type: flying) [18]. Initial respondents underwent the Structured Clinical Interview for DSM-5 (SCID-5) administered by trained professionals. Diagnosis required marked and persistent fear of flying disproportionate to actual danger, immediate anxiety response to flying-related stimuli, active avoidance or intense distress during air travel, significant functional impairment, and symptom persistence for at least six months. The study cohort comprised 60 individuals with confirmed AP, matched 1:1, with 60 FFs serving as controls. FFs were defined as individuals completing a minimum of 25 flights annually and were recruited through parallel advertising strategies. Matching was conducted for both age and sex. Exclusion criteria for both groups included any history of other psychiatric conditions (including anxiety disorders, mood disorders, and substance use disorders), acute or chronic medical conditions, and familial relationships among participants. To encourage participation, subjects were entered into a randomized drawing for ten €50 Amazon gift cards. All study procedures, including clinical interviews and data collection, were conducted in person at our research facility. The study protocol received approval from the local ethics committee (Studio Minoretti; reference number: 2022/10AV), and all participants provided written informed consent.

Personality dimensions

Personality assessment was conducted using the NEO Five-Factor Inventory (NEO-FFI), a validated 60-item instrument designed to measure the fundamental dimensions of personality [19]. This abbreviated version of the full NEO inventory employs a standardized five-point Likert scale methodology, with response options ranging from “totally disagree” to “totally agree,” to evaluate the “big five” personality domains. The instrument’s structure allocates 12 items to each personality dimension, ensuring comprehensive coverage while maintaining assessment efficiency. The NEO-FFI has consistently demonstrated robust psychometric properties, with established internal consistency across its dimensional scales [20]. Longitudinal research has validated the temporal stability of these personality traits, supporting their utility as reliable indicators of enduring individual differences [21].

Covariates

Anthropometric measurements and participants’ general characteristics were collected using standard protocols and questionnaires. BMI was derived using the standard formula of weight in kilograms divided by height in meters squared. Smoking status categorization identified current smokers as individuals reporting lifetime consumption of at least 100 cigarettes and maintaining either daily or intermittent tobacco use [22]. Alcohol consumption patterns were evaluated against sex-specific thresholds, with significant consumption defined as exceeding 20 g (two units) daily for females and 30 g (three units) daily for males [23]. Educational background was quantified through completed years of formal instruction. Physical activity (PA) assessment utilized the International Physical Activity Questionnaire (IPAQ) [24], a standardized self-report tool designed to evaluate PA levels across populations with diverse sociocultural backgrounds. The IPAQ generates activity profiles expressed in metabolic equivalent total (MET) minutes per week, providing a quantifiable measure of energy expenditure associated with PA.

Statistical analysis

As this was an exploratory study, no formal sample size calculation has been performed. The normality of data distribution was assessed using the Kolmogorov-Smirnov test. Continuous variables, which were found to be normally distributed, were summarized as means and standard deviations. To compare the general characteristics of subjects with AP and FFs, the Student’s t-test was employed for continuous variables, whereas chi-square analysis was applied for categorical data. Subsequently, multivariable logistic regression analysis was performed to explore independent associations between the “big five” personality dimensions and the presence of AP. Two models were constructed for this purpose. The first model was adjusted for age and sex, while the second model was fully adjusted, incorporating all potential confounders. Results from these analyses were reported as ORs with their corresponding 95% CIs. All statistical analyses were conducted using IBM SPSS Statistics for Windows, Version 20.0 (Released 2011; IBM Corp., Armonk, NY, USA). Statistical significance was set at a two-tailed p < 0.05.

## Results

Participants’ characteristics

Table 1 presents the demographic and lifestyle characteristics of subjects with AP and FFs. As anticipated, the matching procedures yielded no significant differences in age and sex distribution between the two groups. In addition, BMI, smoking, alcohol consumption, and PA levels in MET minutes per week did not show significant intergroup differences. However, FFs had a significantly higher education level compared to subjects with AP (p < 0.001).

**Table 1:** General characteristics of the study participants Continuous variables are presented as mean ± standard deviation and were compared using independent samples t-tests. Categorical variables are presented as frequencies and were compared using chi-square (χ²) tests. p < 0.001 indicates a statistically significant difference between groups. MET: metabolic equivalent total.

Characteristic	Subjects with aviophobia (n = 60)	Frequent fliers (n = 60)	Test statistic	p-value
Age, years	41.7 ± 3.7	42.1 ± 4.5	t = -0.88	0.38
Sex, male/female	15/45	20/40	χ² = 1.00	0.32
Body mass index, kg/m²	22.4 ± 2.8	22.7 ± 3.0	t = -0.19	0.85
Current smoking, yes/no	8/52	4/56	χ² = 1.48	0.22
Alcohol consumption, yes/no	2/58	1/59	χ² = 0.34	0.56
Education, years	12.1 ± 3.1	14.2 ± 2.9	t = -3.92	<0.001
Physical activity in 1000 MET min/week	3.8 ± 1.9	3.7 ± 1.7	t = 0.36	0.72

Personality traits

In terms of the “big five” personality traits, we found notable differences between the two study groups. Specifically, subjects with AP scored higher on neuroticism (p < 0.001) and lower on conscientiousness (p < 0.001) and agreeableness (p < 0.001) compared to FFs. However, there were no significant intergroup differences with respect to extraversion and openness (Table 2).

**Table 2:** Results of the NEO Five-Factor Inventory for the study sample Values are presented as mean ± standard deviation. Independent samples t-tests were used to compare personality trait scores between subjects with aviophobia and frequent fliers. p < 0.001 indicates statistically significant differences between groups.

"Big five" personality traits	Subjects with aviophobia (n = 60)	Frequent fliers (n = 60)	Test statistic	p-value
Neuroticism	36.2 ± 6.8	30.8 ± 5.9	t = 4.72	<0.001
Extraversion	38.7 ± 6.7	38.3 ± 6.6	t = 0.37	0.71
Conscientiousness	40.7 ± 7.9	45.4 ± 5.9	t = -3.84	<0.001
Agreeableness	37.4 ± 5.2	42.2 ± 4.7	t = -5.39	<0.001
Openness	34.5 ± 5.6	36.4 ± 4.6	t = -1.00	0.32

Multivariable analysis

**Figure 1:**
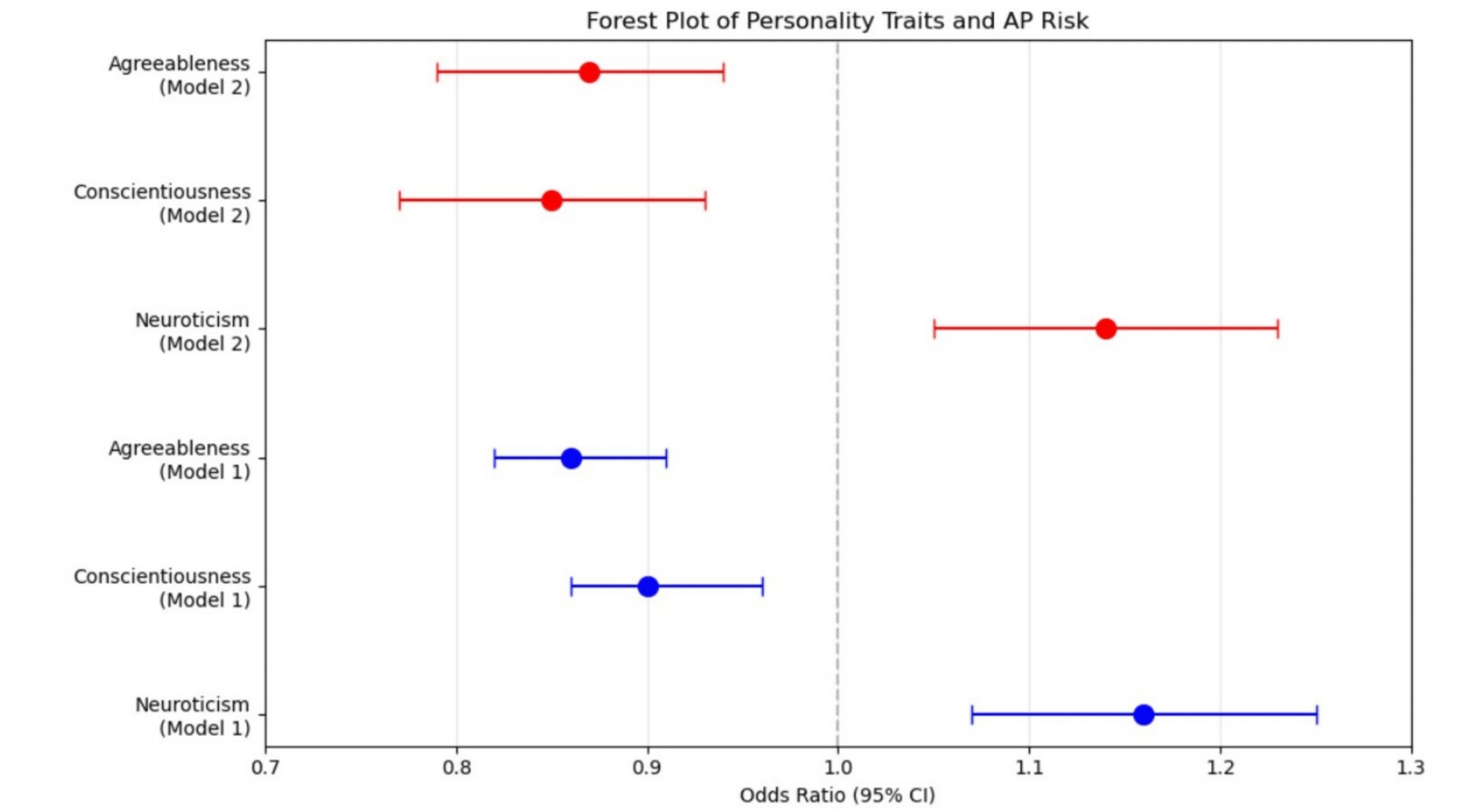
Forest plots showing the association between neuroticism, conscientiousness, and agreeableness and aviophobia risk ORs with 95% CIs are displayed for Model 1 (blue) and Model 2 (red). The vertical dashed line at OR = 1.0 represents no effect. Points to the right of the line indicate increased risk, while points to the left indicate decreased risk.

Multivariable logistic regression analyses revealed independent associations between specific personality dimensions and AP. The first model, adjusted for demographic variables of age and sex, identified three personality traits as independent predictors: elevated neuroticism demonstrated a positive association (OR = 1.16, 95% CI = 1.07-1.25, p < 0.001; i.e., 16% increase in the odds of having AP), while both conscientiousness (OR = 0.90, 95% CI = 0.86-0.96, p < 0.001; i.e., 10% decrease in the odds of having AP) and agreeableness (OR = 0.86, 95% CI = 0.82-0.91, p < 0.001; i.e., 14% decrease in the odds of having AP) exhibited inverse relationships with AP risk. In the second fully adjusted model, these associations maintained their statistical significance following adjustment for additional confounding variables, including BMI, smoking status, alcohol consumption, educational attainment, and PA levels, with minimal modification of effect sizes: neuroticism (OR = 1.14, 95% CI = 1.05-1.23, p < 0.001; i.e., 14% increase in the odds of having AP), conscientiousness (OR = 0.85, 95% CI = 0.77-0.93, p < 0.001; i.e., 15% decrease in the odds of having AP), and agreeableness (OR = 0.87, 95% CI = 0.79-0.94, p < 0.01; i.e., 13% decrease in the odds of having AP). Figure 1 illustrates the associations between the three significant personality dimensions and AP risk through forest plots of adjusted ORs derived from the two multivariable models.

## Discussion

In this study, we identified significant associations between three dimensions of the “big five” personality traits and AP, providing novel insights into the psychological mechanisms underlying flight-related anxiety. Specifically, our results revealed distinct personality profiles among individuals with AP compared to FFs, characterized by increased neuroticism and reduced conscientiousness and agreeableness.

The robust positive association between neuroticism and fear of flying is in accordance with established theoretical models of anxiety disorders. Neuroticism, broadly defined as a predisposition to experience negative emotions such as anxiety, emotional instability, and irritability, is associated with poor stress management and a tendency to perceive ordinary situations as threatening [25]. Our multivariable analysis demonstrated that each unit increase in neuroticism scores was associated with a 14% increase in the odds of having AP. The pervasive and temporally stable nature of neuroticism across the lifespan [25] may partially explain both the high prevalence of AP in the general population and the challenges associated with its treatment. In this scenario, clinically significant episodes of AP may arise from interactions between this personality trait and flight-related stressors, consistent with prior research indicating that individuals with elevated neuroticism exhibit high sensitivity to threat stimuli and increased stress reactivity, predisposing them to specific phobias [17, 25]. Equally noteworthy were the protective effects observed for conscientiousness and agreeableness against AP, which represent novel findings in the context of aviation-related anxiety. The inverse relationship between conscientiousness and AP may reflect the capacity of highly conscientious individuals to employ effective coping strategies and maintain emotional regulation when faced with challenging situations [26,27]. Accordingly, individuals scoring higher on conscientiousness are more likely to exert effort to confront personal fears and tolerate discomfort in perceived threatening contexts [28]. Additionally, conscientiousness has been linked to traits such as achievement striving, dutifulness, and responsibility [29], which may further facilitate adaptive responses to flight-related fears. Conversely, individuals with lower conscientiousness scores may struggle to modify their behavior despite their efforts. Similarly, higher agreeableness has been associated with superior self-control abilities, including the capacity to override cognitive conflicts and intrapsychic urges [30, 31]. This personality dimension may enable individuals to suppress flight-related fears more effectively, thereby reducing their susceptibility to aviation-related anxiety. Together, these findings suggest that both conscientiousness and agreeableness contribute to resilience against flight anxiety through mechanisms involving emotional regulation and cognitive control.

Our findings build upon prior research by Veronese et al. [32], which identified insecure attachment styles as predominant among individuals with AP. The personality traits highlighted in our study may serve as dimensions that underlie these attachment patterns, offering a more nuanced understanding of the psychological mechanisms contributing to flight-related anxiety. Furthermore, our results align with and extend the observations of Laker et al. [9], who documented associations between stress symptoms and aviophobic experiences. This suggests that personality traits may mediate the relationship between stress and AP, providing a potential explanatory framework for this connection. Importantly, the multifaceted influence of the three personality traits examined in our study may also help elucidate the heterogeneity of AP. Accordingly, this condition may encompass a diverse range of fears, including fear of heights (acrophobia), fear of confined or inescapable spaces (claustrophobia), fear of plane crashes, and fear stemming from a perceived lack of control during flight [33]. Such variability underscores the complexity of AP as a psychological phenomenon, which likely arises from an interplay of individual differences in personality, cognitive processes, and situational factors. We believe that the identification of specific personality traits associated with AP may have significant implications for clinical practice. Understanding these relationships could enhance screening procedures by identifying individuals at higher risk for developing AP. Additionally, this knowledge could inform the design of personalized therapeutic interventions within cognitive-behavioral therapy frameworks, particularly those incorporating exposure therapy [4]. In this regard, interventions targeting emotional regulation strategies may be especially beneficial for individuals with elevated neuroticism scores, as this trait is strongly linked to higher sensitivity to stress and threat stimuli [25]. In addition, our findings suggest that addressing maladaptive coping mechanisms associated with low conscientiousness or agreeableness could further improve therapeutic outcomes. For example, strategies aimed at fostering self-discipline and promoting adaptive emotional responses may help mitigate flight-related anxiety in individuals scoring low on conscientiousness. Similarly, enhancing interpersonal trust and cognitive flexibility might benefit those with lower agreeableness scores by reducing their susceptibility to fear-driven avoidance behaviors.

Our findings should be interpreted in the context of certain limitations. First, the case-control design of this study limits our ability to draw causal inferences regarding the relationship between personality traits and the development of AP. To address this caveat, future longitudinal investigations are needed to explore the temporal dynamics of these associations and to examine potential explanatory factors, such as cognitive processing styles and environmental influences. Furthermore, research investigating how personality traits may influence treatment outcomes could yield valuable insights for tailoring clinical interventions. Second, although our sample was well-matched on several key variables, the higher education levels observed among FFs may have influenced the results. While we accounted for this variable in our fully adjusted multivariable model, residual confounding cannot be entirely excluded. Future studies with more demographically balanced samples could help clarify the role of education in shaping these associations. Finally, as this study was conducted exclusively within an Italian population, the generalizability of our findings to other cultural contexts remains uncertain. Cultural differences in attitudes toward air travel and expressions of anxiety may influence the observed relationships between personality traits and AP. Cross-cultural validation is therefore essential to determine whether these associations hold across diverse populations and to identify any culturally specific factors that may impact the development or expression of AP.

## Conclusions

The findings of this case-control study revealed distinct personality profiles between individuals with AP and FFs, characterized by significant differences in three key personality dimensions. People with AP exhibited higher neuroticism scores and lower levels of both conscientiousness and agreeableness. These personality traits remained independently associated with AP even after adjusting for multiple confounding factors, suggesting they play a fundamental role in flight-related anxiety. The identification of these specific personality patterns offers valuable insights for clinical practice by enabling more targeted screening approaches and personalized therapeutic interventions. Understanding how elevated neuroticism may increase susceptibility to flight anxiety, while higher conscientiousness and agreeableness appear protective, can help clinicians develop more effective treatment strategies. This knowledge could enhance cognitive-behavioral therapy approaches by incorporating personality-specific components that address emotional regulation, coping mechanisms, and adaptive responses to flight-related stress. While further research is needed to fully understand these relationships across different populations and cultures, these findings represent an important step forward in our understanding of AP’s psychological underpinnings and potential treatment approaches.

## References

[1] Donker T, Fehribach JR, van Klaveren C, Cornelisz I, Toffolo MB, van Straten A, van Gelder JL (2023). Automated mobile virtual reality cognitive behavior therapy for aviophobia in a natural setting: a randomized controlled trial. Psychol Med.

[2] Fehribach JR, Toffolo MB, Cornelisz I, van Klaveren C, van Straten A, van Gelder JL, Donker T (2021). Virtual reality self-help treatment for aviophobia: protocol for a randomized controlled trial. JMIR Res Protoc.

[3] Busscher B, Spinhoven P, de Geus EJ (2020). Synchronous change in subjective and physiological reactivity during flight as an indicator of treatment outcome for aviophobia: A longitudinal study with 3-year follow-up. J Behav Ther Exp Psychiatry.

[4] Abuso AB, Hashmi M, Hashmi H, Khoo A, Parsaik A (2023). Overcoming fear of flying: a combined approach of psychopharmacology and gradual exposure therapy. Cureus.

[5] Gottlieb A, Doniger GM, Hussein Y, Noy S, Plotnik M (2021). The efficacy of a virtual reality exposure therapy treatment for fear of flying: a retrospective study. Front Psychol.

[6] Ekeberg Ø, Seeberg I, Ellertsen BB (1989). The prevalence of flight anxiety in Norway. Nord J Psychiatry.

[7] Van Gerwen LJ, Diekstra RF (2000). Fear of flying treatment programs for passengers: an international review. Aviat Space Environ Med.

[8] Oakes M, Bor R (2010). The psychology of fear of flying (part II): a critical evaluation of current perspectives on approaches to treatment. Travel Med Infect Dis.

[9] Laker MK, Bob P, Riethof N, Raboch J (2024). Fear of flying, stress and epileptic-like symptoms. Neuropsychiatr Dis Treat.

[10] Fleischer A, Tchetchik A, Toledo T (2012). The impact of fear of flying on travelers’ flight choice: choice model with latent variables. J Travel Res.

[11] McCrae RR, John OP (1992). An introduction to the five-factor model and its applications. J Pers.

[12] Redelmeier DA, Najeeb U, Etchells EE (2021). Understanding patient personality in medical care: five-factor model. J Gen Intern Med.

[13] A Widiger T (2011). Personality and psychopathology. World Psychiatry.

[14] Torgersen S (2011). Personality may be psychopathology, and vice versa. World Psychiatry.

[15] Pélissolo A, André C, Pujol H (2002). Personality dimensions in social phobics with or without depression. Acta Psychiatr Scand.

[16] Capriola NN, Booker JA, Ollendick TH (2017). Profiles of temperament among youth with specific phobias: implications for CBT outcomes. J Abnorm Child Psychol.

[17] Bienvenu OJ, Hettema JM, Neale MC, Prescott CA, Kendler KS (2007). Low extraversion and high neuroticism as indices of genetic and environmental risk for social phobia, agoraphobia, and animal phobia. Am J Psychiatry.

[18] Thng CE, Lim-Ashworth NS, Poh BZ, Lim CG (2020). Recent developments in the intervention of specific phobia among adults: a rapid review. F1000Res.

[19] Rosellini AJ, Brown TA (2011). The NEO Five-Factor Inventory: latent structure and relationships with dimensions of anxiety and depressive disorders in a large clinical sample. Assessment.

[20] Ramanaiah NV, Rielage JK, Cheng Y (2002). Cloninger's temperament and character inventory and the NEO Five-Factor Inventory. Psychol Rep.

[21] Rantanen J, Metsäpelto RL, Feldt T, Pulkkinen L, Kokko K (2007). Long-term stability in the Big Five personality traits in adulthood. Scand J Psychol.

[22] Klein H, Sterk CE, Elifson KW (2014). Smoke and mirrors: the perceived benefits of continued tobacco use among current smokers. Health Psychol Res.

[23] Petroni ML, Brodosi L, Marchignoli F, Musio A, Marchesini G (2019). Moderate alcohol intake in non-alcoholic fatty liver disease: to drink or not to drink?. Nutrients.

[24] Hagströmer M, Oja P, Sjöström M (2006). The International Physical Activity Questionnaire (IPAQ): a study of concurrent and construct validity. Public Health Nutr.

[25] Widiger TA, Oltmanns JR (2017). Neuroticism is a fundamental domain of personality with enormous public health implications. World Psychiatry.

[26] Bartley CE, Roesch SC (2011). Coping with daily stress: the role of conscientiousness. Pers Individ Dif.

[27] Chen L, Qu L, Hong RY (2022). Pathways linking the big five to psychological distress: exploring the mediating roles of stress mindset and coping flexibility. J Clin Med.

[28] Javaras KN, Schaefer SM, van Reekum CM (2012). Conscientiousness predicts greater recovery from negative emotion. Emotion.

[29] Sutin AR, Aschwanden D, Stephan Y, Terracciano A (2022). The association between facets of conscientiousness and performance-based and informant-rated cognition, affect, and activities in older adults. J Pers.

[30] Ode S, Robinson MD (2007). Agreeableness and the self-regulation of negative affect: findings involving the neuroticism/somatic distress relationship. Pers Individ Dif.

[31] Laursen B, Pulkkinen L, Adams R (2002). The antecedents and correlates of agreeableness in adulthood. Dev Psychol.

[32] Veronese G, Procaccia R, Romaioli D, Barola G, Castiglioni M (2013). Psychopathological organizations and attachment styles in patients with fear of flying: a case study. Open Psychol J.

[33] Clark GI, Rock AJ (2016). Processes contributing to the maintenance of flying phobia: a narrative review. Front Psychol.

